# Comment on Indoxyl Sulfate—Review of Toxicity and Therapeutic Strategies. *Toxins* 2016, *8*, 358

**DOI:** 10.3390/toxins9040142

**Published:** 2017-04-17

**Authors:** Fellype C. Barreto, Daniela V. Barreto, Andrea E. M. Stinghen, Ziad A. Massy

**Affiliations:** 1 Division of Nephrology, Department of Internal Medicine, Federal University of Paraná, 80060-900 Curitiba, Brazil; danielaveitbarreto@gmail.com; 2 Experimental Nephrology Laboratory, Basic Pathology Department, Federal University of Paraná, 81531-980 Curitiba, Brazil; andreastinghen@ufpr.br; 3 Division of Nephrology, Ambroise Paré University Hospital, APHP, Boulogne-Billancourt, Paris 92104, France; ziad.massy@aphp.fr; 4 INSERM Unit 1018, CESP, University of Versailles-Saint-Quentin-en-Yvelines, University Paris-Saclay, 54500 Villejuif, France

**Keywords:** uremic toxin, indoxyl sulfate, bone disease

## Abstract

Recently, the clinical and experimental evidences that support the toxic effects of indoxyl sulfate, a protein-bound uremic toxin in chronic kidney disease (CKD) patients, has been discussed. In this panorama, the authors described several in vitro and in vivo studies, suggesting that indoxyl sulfate may play a part in the pathogenesis of low turnover bone disease. However, the discussion claims the need for relevant clinical studies in CKD patients whose bone turnover biomarkers and bone histomorphometry were assessed in order to demonstrate the association between serum levels of indoxyl sulfate and bone turnover. We would like to underline the availability of this clinical data to support the concept that indoxyl sulfate may play a part in the pathogenesis of low turnover bone disease in CKD patients.

In a recent review article on the toxicity of indoxyl sulfate, Leong and Sirich [1] discussed the clinical and experimental evidences that support the toxic effects of this protein-bound uremic toxin in chronic kidney disease (CKD) patients. We wish to comment on the bone disease topic that, in our opinion, provides misinformation. As correctly discussed by the authors, several in vitro and in vivo studies have suggested that indoxyl sulfate may play a part in the pathogenesis of low turnover bone disease as demonstrated by its effects on: (i) reducing parathyroid hormone (PTH) receptor expression on osteoblasts; (ii) inhibiting osteoclast differentiation and function; and (iii) reducing bone turnover in an animal model of CKD [2,3,4]. We would like, contrary to the information provided by the authors, to extend this information to include available clinical studies that have assessed the association of indoxyl sulfate and bone disease including low turnover bone disease in CKD patients.

In a study published in 2010, Goto et al. [5] investigated in a cohort of 47 hemodialysis patients the association between serum levels of indoxyl sulfate and biomarkers of bone turnover, named alkaline phosphatase, bone-specific alkaline phosphatase (both markers of bone formation) and tartrate-resistant acid phosphatase 5b (marker of bone resorption). Indoxyl sulfate was negatively associated with bone formation markers, independently of PTH levels, giving further support to the concept that indoxyl sulfate may play a part in the skeletal resistance to PTH observed in CKD. This was the first study to investigate the association between indoxyl sulfate and bone disease in the clinical setting, but was limited to the evaluation of serum markers of bone turnover.

More recently, in 2014, Barreto et al. [6] have assessed the association between indoxyl sulfate serum levels, serum markers of bone turnover and bone histomorphometry in a cohort of 49 pre-dialysis (stages 2 from 5) CKD patients. Bone histomorphometry is considered the gold standard method for diagnosing bone disease, including renal osteodysthrophy, and may provide accurate information on bone microarchitecture and dynamic parameters of bone formation [7]. A positive association was noted between indoxyl sulfate and bone histomorphometry parameters, such as bone formation rate, osteoid volume and osteoblast surface. Most patients had a very low bone formation rate, characteristic of low turnover bone disease. These somewhat unexpected direct associations do not invalidate previous in vitro studies that pointed to a possible role of indoxyl sulfate on the pathogenesis of low bone turnover disease, though. One possible explanation for this apparent paradox is that at the early stages of CKD the inhibitory actions of high indoxyl sulfate levels might overcome the stimulatory effect of PTH, because this hormone levels are only slightly elevated as in pre-dialysis patients. Other important lesson from this study is that it draws attention to one important fact: is not always an easy task to translate experimental studies generated hypothesis. Unlike experimental models, clinical studies, particularly in the CKD setting, must deal with the interaction a variety of confounding circulating factors that may affect homeostasis, including bone metabolism. As a result, in the real life setting, one can hardly ever determine an isolated culprit for a given pathological condition [8].

To the best of our knowledge, these are, so far, the studies that have evaluated the association between indoxyl sulfate and bone disease. Clinical studies are the Cinderella to generate and to prove hypothesis as well as to validate new healthcare technologies. We hope that we are able to extend the experimental data reported by Leong and Sirich to include available clinical data to support the concept that uremic toxins such as indoxyl sulfate may play a part in the pathogenesis of low turnover bone disease in CKD patients [8]. 
